# Submicroscopic 16q24.2–q24.3 deletion in a family with nonsyndromic short stature

**DOI:** 10.1038/s41439-026-00336-4

**Published:** 2026-01-26

**Authors:** Chisato Narita, Hidekazu Utsunomiya, Junpei Hamada, Ikuko Kageyama, Maki Fukami, Akie Nakamura

**Affiliations:** 1 Department of Molecular Endocrinology, National Research Institute for Child Health and Development, Tokyo, Japan; 2 Department of Pediatrics and Adolescent Medicine, Juntendo University Graduate School of Medicine, Tokyo, Japan; 3 Department of Pediatrics, Ehime University Graduate School of Medicine, Ehime, Japan

**Keywords:** Growth disorders, Clinical genetics

## Abstract

Array-based comparative genomic hybridization for a boy, his mother and his half-sister with etiology-unknown nonsyndromic short stature identified a hitherto unreported heterozygous ~1.5-Mb deletion at 16q24.2–q24.3. Whole-exome sequencing detected no pathogenic variants. Our results, in conjunction with previous reports of cases with similar deletions, indicate that the 16q24.2–q24.3 region provides a platform for submicroscopic deletions and possibly contains a gene(s) or regulatory elements involved in skeletal growth.

Nonsyndromic short stature is a relatively common disorder caused by a combination of genetic and environmental factors^1^. Loss-of-function variants in *ACAN*, *SHOX*, *NPR2* and some other genes have been implicated in nonsyndromic short stature^2,3^. However, these monogenic mutations explain less than 20% of cases^4,5^, indicating that other genetic factors play important roles in the development of this disorder. In this context, submicroscopic chromosomal deletions have been identified in several patients with nonsyndromic short stature, although these abnormalities are more commonly associated with syndromic short stature^6–9^.

Here, we report three individuals from the same family who presented with nonsyndromic short stature and carried a submicroscopic deletion at 16q24.2–q24.3. The proband (Fig. 1A, II-5) was a 1-year-old Japanese boy. He was born at 36 weeks and 3 days of gestation with normal birth length and weight of 48.0 cm (+0.64 s.d.) and 2,474 g (−0.41 s.d.), respectively. At 1 year of age, he was referred to our hospital for the evaluation of short stature. He manifested short stature (69.1 cm, −2.27 s.d.), with normal weight (9.5 kg, +0.12 s.d.). No dysmorphic features or congenital anomalies were noted on physical examination. Laboratory tests revealed age-appropriate levels of thyroid hormones, gonadotropins and sex hormones. His insulin-like growth factor 1 level was within the normal range (21 ng/ml, −1.09 s.d.). A whole-body skeletal X-ray survey showed no remarkable findings, excluding skeletal dysplasia (Fig. 1B). He was diagnosed with nonsyndromic short stature and was followed up without medical intervention. Subsequently, his height increased along the −2.0 or −2.5 s.d. growth curves for Japanese boys (Fig. 1C). His developmental milestones were almost age-appropriate, although he was noted to have slightly delayed speech at 2 years and 8 months. During the follow-up period, he showed no clinical abnormalities except for recurrent otitis media. His mother (I-3) showed severe short stature of 130 cm (−5.36 s.d.) and spina bifida. Detailed clinical records of this individual were unavailable. A half-sister of the proband (II-1) also showed severe short stature (135 cm, −4.40 s.d.). She also had ventricular septal defect (VSD). Allegedly, a growth hormone provocation test during her childhood yielded normal results. No additional clinical features, such as intellectual disability or dysmorphic facial appearance, were noted in the mother or the half-sister. The other members of this family were phenotypically normal, except for a brother of the proband (II-4) who had mild short stature (−2.61 s.d.). Clinical information about the brother was unavailable.

**Fig. 1:**
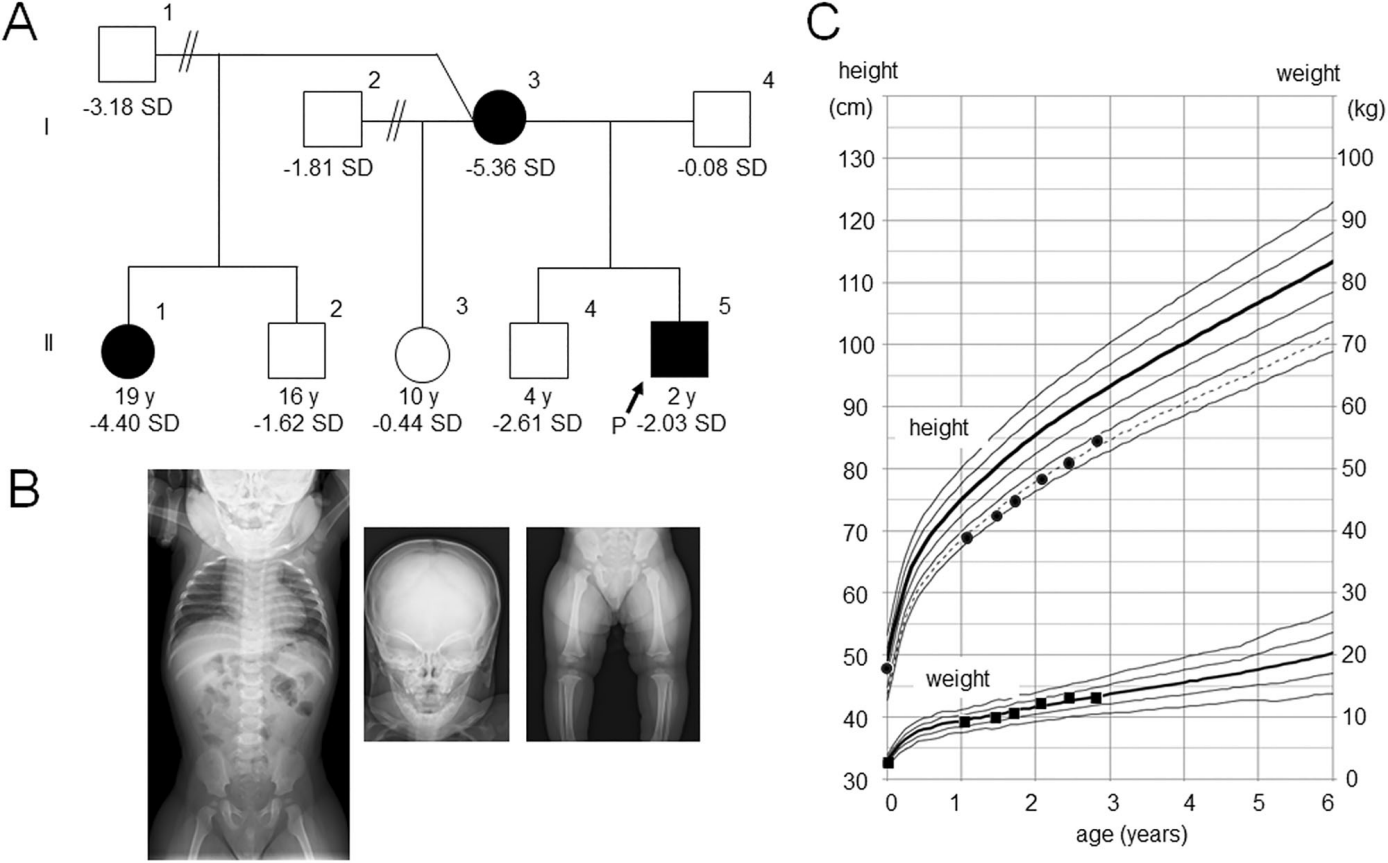
Clinical features of the family. **A** The pedigree of the family. The black square and circles indicate individuals with nonsyndromic short stature and 16q24.2-q24.3 deletion. **B** Whole-body X-ray of the proband at 1 year of age. **C** Growth chart of the proband.

Genomic DNA samples were extracted from peripheral leukocytes of the proband (II-5), the mother (I-3) and the half-sister (II-1). Samples of other family members were unavailable for genetic analysis. First, we conducted whole-exome sequencing as described previously^10^. The results showed no nucleotide changes that could explain the short stature of the three individuals. Then, we performed comparative genomic hybridization using a catalog array (Sure Print G3, 4 × 180K format, Agilent Technologies). As a result, we identified a heterozygous deletion in the 16q24.2–q24.3 region in the three patients. The deletion was ~1.5 Mb in size (maximum, Chr16: 87,800,426–89,304,429; minimum, Chr16: 87,800,485–89,304,370; GRCh37/hg19) and encompassed 23 protein-coding genes (Fig. 2). The deletion was not registered in the Database of Genomic Variants (https://dgv.tcag.ca/dgv/app/home) or gnomAD (https://gnomad.broadinstitute.org/). Fourteen of the 23 affected genes have been linked to human disorders (Supplementary Table 1). In particular, *CDT1*, *GALNS* and *PIEZO1* were reported to cause syndromic short stature in an autosomal recessive manner (Supplementary Table 1). Furthermore, genetic knockout of *Znf469* and *Trappc2l* was associated with decreased body length in mice (International Mouse Phenotyping Consortium; https://www.mousephenotype.org/). However, none of these genes is known to cause autosomal dominant short stature in humans. In addition, *ANKRD11*, a causative gene for autosomal dominant KBG syndrome characterized by short stature, recurrent otitis, epilepsy, heart anomalies and skeletal anomalies^11,12^, was located at a position ~29 kb from the deletion. However, Hi-C and Micro-C data of H1 human embryonic stem cells in the UCSC Genome Browser (https://genome.ucsc.edu/) suggested no close interaction between the deleted region and *ANKRD11* (Supplementary Fig. 1).

**Fig. 2:**
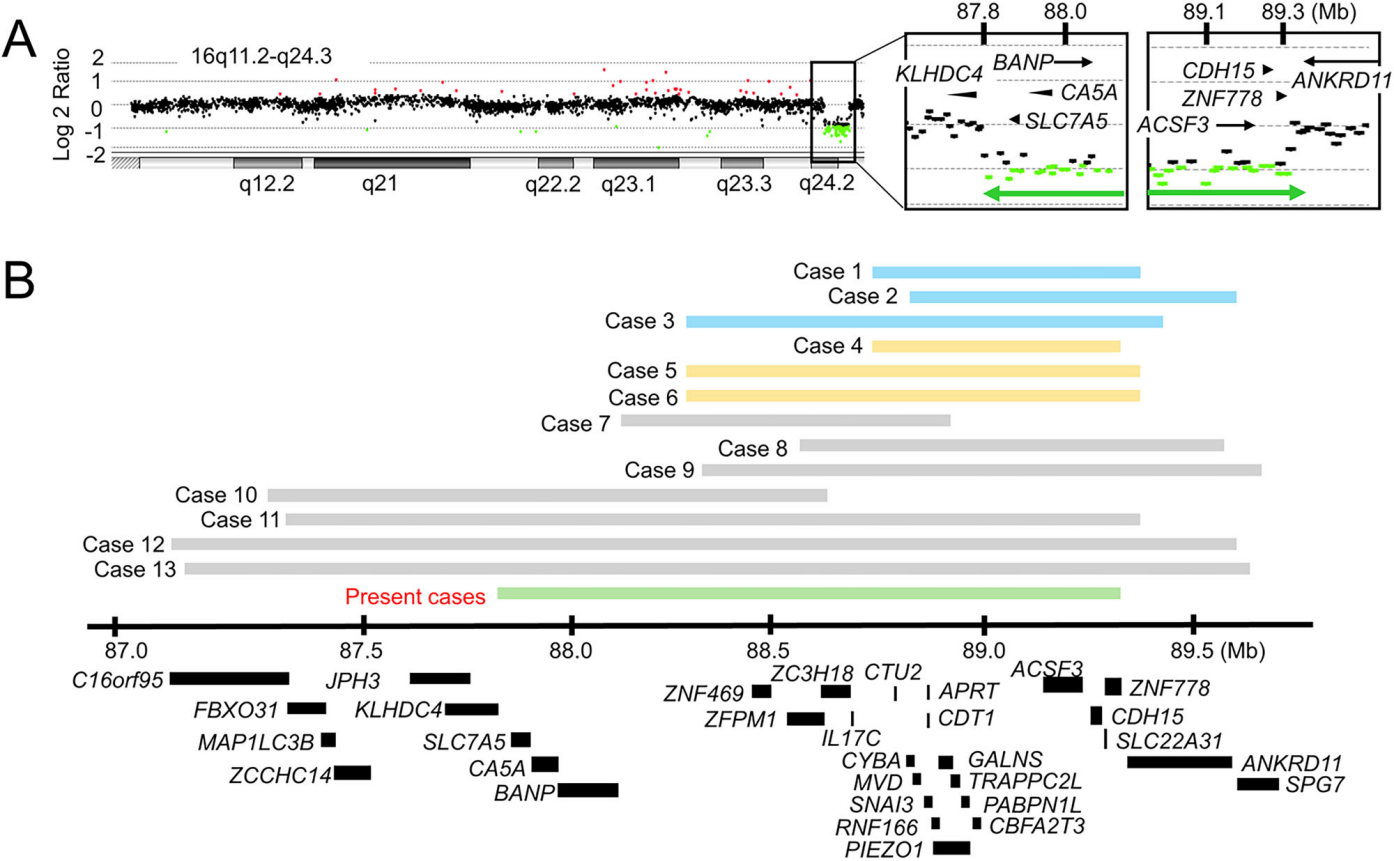
Deletions identified in the present and previously reported cases. **A** Array-based comparative genomic hybridization for the 16q11.2–q24.3 region in our cases. Green dots depict the copy-number loss region (log_2_ ratio of <−0.9), while black dots denote copy-number neutral regions. The genomic positions are based on GRCh37/hg19. The positions of protein-coding genes are shown. **B** Deletions in the present and known cases. Cases 1–13 were reported previously^6–9^. Blue and yellow lines indicate the deletions of individuals with and without short stature, respectively. Gray lines depict the deletions of individuals whose height data were unavailable.

Next, we consulted previous reports of deletions at 16q24.2–q24.3. Thus far, submicroscopic deletions in this region have been identified in 13 cases^6–9^ (Fig. 2B). The sizes of the deletions ranged from 690 kb to ~2.30 Mb. Most of the 13 patients exhibited dysmorphic features and intellectual disability with some additional clinical symptoms (Supplementary Table 2). VSD and multiple skeletal anomalies were observed in one case each (cases 7 and 12, respectively). Three of the 13 patients (cases 1–3) were reported to have short stature, while 3 had normal stature (cases 4–6), and height data were unavailable for the remaining 7 cases. We observed no apparent genotype–phenotype correlation in the 13 cases, except for the relatively severe phenotype in case 12 with a large deletion.

This study provided two notable findings. First, our data, in conjunction with prior observations^6–9^, imply that the ~2-Mb genomic interval at 16q24.2–q24.3 represents a platform for submicroscopic deletions. Because the present and previous deletions had different breakpoints (Fig. 2B), these abnormalities are more likely to arise from replication-based errors or nonhomologous end joining than non-allelic homologous recombination^13^. The genomic interval at 16q24.2–q24.3 may contain elements that facilitate de novo deletion, because it is known that de novo copy-number variants in the human genome tend to be clustered in regions with specific chromosomal architectural features^14^. Notably, the deleted interval harbors several genes implicated in congenital malformations or intellectual disability (Supplementary Table 1). Consistent with this, previously reported 13 cases manifested intellectual disability with additional clinical features (Supplementary Table 2). Furthermore, VSD of our patient (II-1) and case 7 can be ascribed to the haploinsufficiency of *ZFPM1* and/or *ZNF778*^9^. While the proband of this study exhibited no apparent congenital malformations or intellectual disability, this can be explained by the broad phenotypic variations in patients with heterozygous submicroscopic deletions^6–9^. Second, in the present family, the etiological relationship between the ~1.5-Mb deletion and nonsyndromic short stature remains uncertain. Because we could not obtain genomic DNA samples from three siblings of the proband, it was impossible to confirm the segregation between the deletion and short stature in this family. However, the deletion was the sole genetic abnormality identified in the three patients by whole-exome sequencing and array-based comparative genomic hybridization. Considering that at least 3 of the 13 known cases (cases 1–3) exhibited short stature (Fig. 2 and Supplementary Table 2), this region probably contains a gene(s) involved in skeletal growth. In this regard, the functionally uncharacterized genes *BANP* and *ZC3H18* may play important roles in human health, because these genes are ubiquitously expressed and have high probability of loss-of-function intolerance (pLI) scores indicative of functional importance (Supplementary Table 1). Alternatively, the short stature in our cases may result from dysregulation of a nearby gene. Although Hi-C and Micro-C data suggested no close interaction between the deleted region and *ANKRD11*, we cannot exclude the possibility that the deletion encompasses a distal enhancer of *ANKRD11*. Further studies are necessary to clarify the etiological relationship between 16q24.2–q24.3 deletions and short stature.

In summary, we identified a hitherto unreported ~1.5-Mb deletion in a family with nonsyndromic short stature. The results of this study, in conjunction with those of previous studies, indicate that the genomic region at 16q24.2–q24.3 is prone to submicroscopic deletions and possibly contains a gene(s) or regulatory elements involved in skeletal growth. Our findings need to be validated in future studies.

## HGV Database

The relevant data from this Data Report are hosted at the Human Genome Variation Database at 10.6084/m9.figshare.hgv.3595.

## Supplementary information

Supplementary Information
