# Supplementary material for: Submicroscopic 16q24.2–q24.3 deletion in a family with nonsyndromic short stature
Source: Hum Genome Var. 2026 Jan 26;13:2. doi: 10.1038/s41439-026-00336-4 (PMC12855966; doi:10.1038/s41439-026-00336-4)
Supplement: Supplementary file 1 — Supplementary Information [file 41439_2026_336_MOESM1_ESM.pdf]

Supplementary Information for

Submicroscopic 16q24.2-q24.3 deletion in a family with non-syndromic short stature

Chisato Narita, Hidekazu Utsunomiya, Junpei Hamada, Ikuko Kageyama, Maki Fukami*, and Akie Nakamura

*Corresponding Author: Maki Fukami
Department of Molecular Endocrinology, National Research Institute for Child Health and Development, 2-10-1 Okura, Setagaya, Tokyo 157-8535, Japan
Tel: (+81) 3 5494 7120 7745 ; Fax: (+81) 3 5494 7026
E-mail: fukami-m@ncchd.go.jp

The Supplementary Information includes two tables and one figure provided as PDF files.

Supplementary Table 1. Protein-coding genes in the deletion.

Supplementary Table 1 lists 23 protein-coding genes located within the deletion of the present cases. The table includes information on expression sites, associated human phenotypes, inheritance patterns, probability of loss-of-function intolerance (pLI) scores, and knockout mouse phenotypes for each gene.

Supplementary Table 2. Previously reported cases with deletions at 16q24.2-q24.3.

Supplementary Table 2 summarizes molecular and clinical data of previously reported cases with deletions at 16q24.2-q24.3. Of the 13 cases, three (cases 1-3) presented with short stature, while three (cases 4–6) had normal height. Height data were unavailable for the remaining cases.

Supplementary Fig. 1. Chromosome interaction landscape of the 16q24.2–q24.3 region involving the deletion and *ANKRD11*.

Hi-C and Micro-C data of H1 human embryonic stem cells are shown.

Supplementary Table 1. Protein-coding genes in the deletion

name	ID	expression site	human phenotype	inheritance	pLI score[a]	knockout mouse phenotype
SLC7A5	NM_003486.7	testis, skeletal muscle	neurodevelopmental disorder	AR	0.24	preweaning lethality
CA5A	NM_001739.2	liver	hyperammonemia	AR	0	no data
BANP	NM_017869.4	ubiquitous	no data		0.99	decreased bone mineral content
ZNF469	NM_001367624.2	ovary	brittle cornea syndrome 1, scoliosis	AR	0.72	abnormal auditory brainstem response, decreased body length
ZFPM1	NM_153813.3	ubiquitous	congenital heart disease	AD	0.24	no data
ZC3H18	NM_144604.4	ubiquitous	no data		1.00	no data
IL17C	NM_013278.4	testis, brain	no data		0	no data
CYBA	NM_000101.4	whole blood, spleen	granulomatous disease	AR	0	increased leukocyte cell number
MVD	NM_002461.3	brain, skeletal muscle	porokeratosis 7	AD	0	abnormal vocalization
SNAI3	NM_178310.4	whole blood, spleen	no data		0.01	no data
RNF166	NM_178841.4	whole blood, spleen	no data		0.49	hyperactivity
CTU2	NM_001012759.3	uterus, testis, skeletal muscle	microcephaly, facial dysmorphism, renal agenesis, ambiguous genitalia, intrauterine growth restriction	AR	0	increased basophil/eosinophil cell number
PIEZO1	NM_001142864.4	sigmoid colon, lung, skeletal muscle	lymphatic malformation 6, dehydrated hereditary stomatocytosis, short stature	AD, AR	0	abnormal limb bud morphology, embryonic growth retardation
CDT1	NM_030928.4	esophagus, testis	Meier-Gorlin syndrome 4 (short stature, small external ears, and reduced or absent patellae)	AR	0	no data
APRT	NM_000485.3	skin, skeletal muscle	adenine phosphoribosyl transferase deficiency	AR	0	preweaning lethality
GALNS	NM_000512.5	testis, skeletal muscle	mucopolysaccharidosis type 4A, short stature	AR	0	no data
TRAPPC2L	NM_016209.5	testis, skeletal muscle, adrenal gland	encephalopathy	AR	0	abnormal bone structure, decreased body length
PABPN1L	NM_001080487.4	brain	no data		0	female infertility
CBFA2T3	NM_005187.6	brain	no data		0.05	no data
ACSF3	NM_174917.5	ubiquitous	combined malonic and methylmalonic acidemia	AR	0	increased circulating iron level
CDH15	NM_004933.3	brain	intellectual impairment	AD	0	no data
SLC22A31	NM_001366322.1	brain, skeletal muscle	no data		0	no data
ZNF778	NM_182531.5	ubiquitous	no data		0	no data

AR: autosomal recessive; AD: autosomal dominant;

[a]pLI, probability of loss-of-function intolerance (https://gnomad.broadinstitute.org/help/constraint). Values > 0.9 indicate intolerance to loss-of-function variants.

Supplementary Table 2. Previously reported cases with deletions at 16q24.2-q24.3

Case	Deletion			Salient clinical features of the patient		Refernece[b,c]
	Affected region[a]	Estimated size	Genes in deletion	Clinical features	Height	
Patients with short stature						
1	88,697,738-89,363,602 (bp)	665.87 kb	*ZC3H18, IL17C, CYBA, MVD, SNAI3, RNF166, CTU2, PIEZO1, CDT1, APRT, GALNS, TRAPPC2L, PABPN1L, CBFA2T3, ACSF3, CDH15, SLC22A31, ZNF778, ANKRD11*	facial dysmorphism, atypical behavior, intellectual disability	short stature (no actual data)	Decipher (299919)
2	88,755,312-89,584,412 (bp)	829 kb	*RNF166, CTU2, PIEZO1, CDT1, APRT, GALNS, TRAPPC2L, PABPN1L, CBFA2T3, ACSF3, CDH15, SLC22A31, ZNF778, ANKRD11, SPG7*	intellectual disability	<-2 SD	Decipher (265435) Patient 4 in reference 9
3	88.23-89.39 (Mb)	1.16 Mb	*ZNF469, ZFPM1, ZC3H18, IL17C, CYBA, MVD, SNAI3, RNF166, CTU2, PIEZO1, CDT1, APRT, GALNS, TRAPPC2L, PABPN1L, CBFA2T3, ACSF3, CDH15, SLC22A31, ZNF778, ANKRD11*	facial dysmorphism, intellectual disability, seizure	<-2 SD	Patient 13 in reference 6
Patients with normal stature						
4	88,641,808–89,332,049 (bp)	690 kb	*ANKRD11* , 17 other genes	facial dysmorphism, brachydactyly, intellectual disability	-1.4 SD	reference 7
5	88.23–89.36 (Mb)	1.1 Mb	*ANKRD11* , 20 other genes	facial dysmorphism, autism spectrum disorder	10-25th percentile	Patient 4 in reference 8
6	88,230,760-89,363,742 (bp)	1.133 Mb	*ANKRD11* , 20 other genes	facial dysmorphism, astigmatism, intellectual disability	-1 SD	ClinVar (SCV000328247) Patient 7 in reference 9
Patients whose height data were unavairable						
7	88,165,980-88,914,268 (bp)	748 kb	*ZNF469, ZFPM1, ZC3H18, IL17C, CYBA, MVD, SNAI3, RNF166, CTU2, PIEZO1, CDT1, APRT, GALNS*	facial dysmorphism, speech delay, ventricular septal defect	no data	ClinVar (SCV000328246) Patient 6 in reference 9
8	88,556,191-89,557,911 (bp)	1 Mb	*ANKRD11* , 19 other genes	genital abnormality, frontal bossing, intellectual disability	no data	Decipher (251801) Patient 5 in reference 9
9	88,335,976-89,603,390 (bp)	1.23 Mb	*ANKRD11* , 21 other genes	intellectual disability	no data	Decipher (290475)
10	87,319,450-88,669,353 (bp)	1.32 Mb	*ZNF469, FBXO31, KLHDC4, CA5A, JPH3, BANP, C16orf95, ZC3H18, SLC7A5, ZCCHC14, MAP1LC3B, ZFPM1*	facial dysmorphism, generalized hypotonia, thoracic hypoplasia	no data	Decipher (260745)
11	87,340,135-89,335,428 (bp)	1.99 Mb	*ANKRD11* , 29 other genes	obesity, facial dysmorphism, synophrys,short foot, shortpalm, macrocephaly, intellectual disability	<-1 SD (no actual data)	Decipher (255327) Patient 2 in reference 9
12	87,183,661-89,520,803 (bp)	2.30 Mb	*ANKRD11* , 29 other genes	congenital thrombocytopenia, ptosis,kidney abnormality,mild intrauterine growth retardation, facial dysmorphism, multiple skeletal anomalies, intellectual disability	no data	Decipher (289169)
13	87,219,866-89,561,087 (bp)	2.31 Mb	*ANKRD11* , 29 other genes	facial dysmorphism, rocker bottom foot, prominent metopic ridge, intellectual disability	no data	Decipher (321856)

[a]The genomic positions refer to the human reference genome (GRCh37/hg19).

[b]Decipher (https://www.deciphergenomics.org/)

[c]ClinVar (https://www.ncbi.nlm.nih.gov/clinvar/)

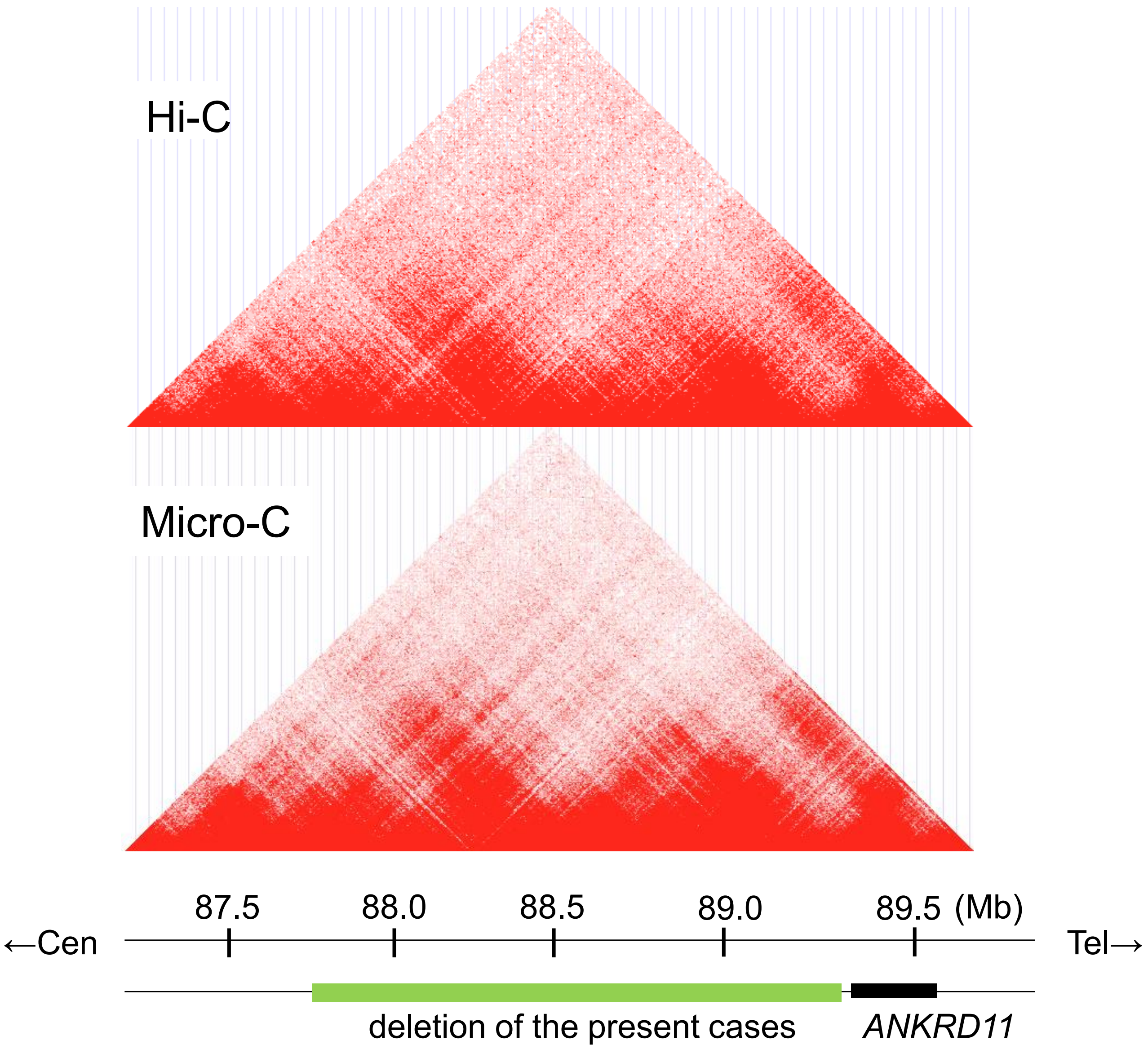
Hi-C
Micro-C
87.5
88.0
88.5
89.0
89.5
(Mb)
←Cen
Tel→
deletion of the present cases
ANKRD11
